# Benzodiazepine use during cariprazine treatment in acute schizophrenia

**DOI:** 10.1192/j.eurpsy.2022.283

**Published:** 2022-09-01

**Authors:** C. Correll, B. Sebe, R. Csehi, K. Acsai, Á. Barabássy

**Affiliations:** 1 The Zucker Hillside Hospital, Department Of Psychiatry, Glen Oaks, United States of America; 2 Gedeon Richter Plc, Medical Division, Budapest, Hungary

**Keywords:** benzodiazepine, cariprazine, schizophrénia, Pharmacotherapy

## Abstract

**Introduction:**

Although antipsychotics are first-line treatments for schizophrenia, benzodiazepines (BZDs) are often used as concomitant medications in acutely exacerbated patients due to their anxiolytic and sedative effects. Cariprazine (CAR), a D3-preferring dopamine D2/D3 partial agonist antipsychotic, has been examined in many clinical studies for the treatment of acute schizophrenia, with and without benzodiazepines.

**Objectives:**

To delineate the effects of benzodiazepine-use during cariprazine treatment in acute schizophrenia.

**Methods:**

Pooled data of cariprazine-treated (1.5-6mg/day) and placebo-treated patients from four short-term, randomised, double-blind trials (NCT00404573, NCT01104766, NCT01104779, NCT00694707) were analysed. Baseline characteristics (age, duration of illness) and efficacy outcome parameters (Total and Hostility Factor Score of the Positive and Negative Syndrome Scale [PANSS]) were compared in patients receiving benzodiazepines (for more ≥3 consecutive days) and not receiving benzodiazepines (<3 consecutive days).

**Results:**

Altogether, 36.7% and 40.7% of the CAR-treated and PBO-treated patients required BZDs. BZD-taking was associated with a higher age in both the CAR-treated (p=0.0002) and PBO-treated (p<0.0001) patients, and with longer illness-duration in both treatment groups (p<0.0001). PANSS Total Score at baseline was similar for BZD users and non-users (CAR: LS Mean=96.36 and 96.27; PBO: LS Mean=95.55 and 96.66). Change from baseline in the PANSS Total Score was greater for patients who did not use BZD vs those who did (CAR: LS Mean= -23.8 vs LS Mean 17.2, p<0.0001; PBO: LS Mean= -14.0 vs LS Mean 12.9, p=0.5776).

**Conclusions:**

These findings may suggest that requiring benzodiazepines is a potential indicator of longer illness duration and poorer response in acute schizophrenia.

**Disclosure:**

I am an employee of Gedeon Richter Plc.

